# Radiomics Analysis for Predicting Epilepsy in Patients With Unruptured Brain Arteriovenous Malformations

**DOI:** 10.3389/fneur.2021.767165

**Published:** 2021-12-15

**Authors:** Shaozhi Zhao, Qi Zhao, Yuming Jiao, Hao Li, Jiancong Weng, Ran Huo, Jie Wang, Hongyuan Xu, Junze Zhang, Yan Li, Zhenzhou Wu, Shuo Wang, Yong Cao, Jizong Zhao

**Affiliations:** ^1^Department of Neurosurgery, Beijing Tiantan Hospital, Capital Medical University, Beijing, China; ^2^China National Clinical Research Center for Neurological Diseases, Beijing, China

**Keywords:** brain arteriovenous malformations, radiomics analysis, epilepsy, machine learning, time-of-flight magnetic resonance angiography

## Abstract

**Objectives:** To investigate the association between radiomics features and epilepsy in patients with unruptured brain arteriovenous malformations (bAVMs) and to develop a prediction model based on radiomics features and clinical characteristics for bAVM-related epilepsy.

**Methods:** This retrospective study enrolled 176 patients with unruptured bAVMs. After manual lesion segmentation, a total of 858 radiomics features were extracted from time-of-flight magnetic resonance angiography (TOF-MRA). A radiomics model was constructed, and a radiomics score was calculated. Meanwhile, the demographic and angioarchitectural characteristics of patients were assessed to build a clinical model. Incorporating the radiomics score and independent clinical risk factors, a combined model was constructed. The performance of the models was assessed with respect to discrimination, calibration, and clinical usefulness.

**Results:** The clinical model incorporating 3 clinical features had an area under the curve (AUC) of 0.71. Fifteen radiomics features were used to build the radiomics model, which had a higher AUC of 0.78. Incorporating the radiomics score and clinical risk factors, the combined model showed a favorable discrimination ability and calibration, with an AUC of 0.82. Decision curve analysis (DCA) demonstrated that the combined model outperformed the clinical model and radiomics model in terms of clinical usefulness.

**Conclusions:** The radiomics features extracted from TOF-MRA were associated with epilepsy in patients with unruptured bAVMs. The radiomics-clinical nomogram, which was constructed based on the model incorporating the radiomics score and clinical features, showed favorable predictive efficacy for bAVM-related epilepsy.

## Introduction

Brain arteriovenous malformations (bAVMs) are tangles of malformed vessels without capillary networks and commonly present with intracranial hemorrhage, epilepsy, or neurological deficits (1, 2). Although hemorrhage prevention is the primary aim of bAVM treatment, seizure control should also be at the forefront of therapeutic management (3). The accurate prediction of epilepsy may open novel therapeutic possibilities for patients with bAVMs. Patients can present initially with epilepsy or experience epilepsy after intracranial hemorrhage (4). Although hemorrhage history has long been considered a strong predictor for bAVM-related epilepsy (5), this factor is not applicable for unruptured bAVMs. Additionally, epilepsy caused by the rupture of bAVMs may affect the identification of factors that correlate with epilepsy in patients without hemorrhage (6). A reliable tool to predict epilepsy specific to patients with unruptured bAVMs is needed for clinical decision-making.

Radiomics is a research branch in the field of medical imaging. Much lesion information that is not recognized by the human eye remains unmined. Radiomics can extract high-dimensional radiomic features from medical images to fully exploit the in-depth information of lesions (7, 8). Based on the rapid development of machine learning and image processing techniques, radiomic analyses have been successfully applied in epilepsy prediction (9, 10). Considering the potential of radiomics analysis to provide a more accurate prediction for bAVM-related epilepsy, the development of a prediction model combining clinical and quantitative imaging features is a necessary step in enhancing care and treatment for patients with unruptured bAVMs.

Time-of-flight magnetic resonance angiography (TOF-MRA) has been regarded as a first-line non-invasive diagnostic tool in the evaluation of cerebrovascular diseases. It is frequently one of the first examinations performed when there is an initial diagnosis of bAVMs (11, 12). TOF-MRA is easy to perform and provides satisfactory images with the simultaneous presentation of vessels and brain tissues. In our study, a total of 176 patients with bAVMs who underwent TOF-MRA imaging were enrolled. We sought to analyze and explore the performance of clinical factors and radiomics features extracted from TOF-MRA in predicting bAVM-related epilepsy. Then, we built and validated a radiomics-clinical nomogram as a useful clinical tool for the individualized preoperative prediction of epilepsy in patients with unruptured bAVMs.

## Methods

### Patients

**Figure 1:**
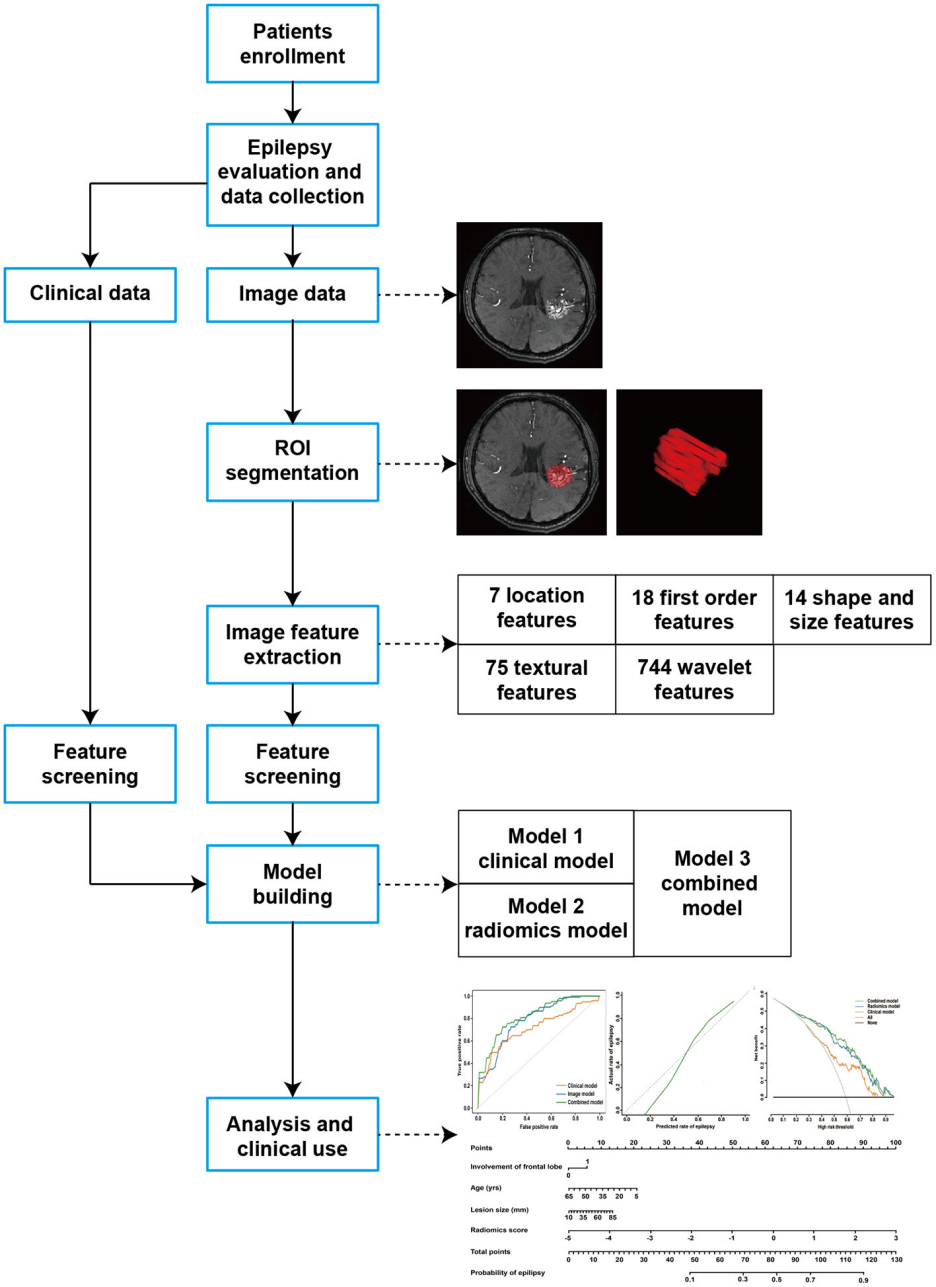
The workflow of the study.

A total of 176 patients with unruptured bAVMs were retrospectively reviewed from our bAVM database of two prospective clinical trials (ClinicalTrials.gov Identifier: NCT01758211 and NCT02868008) between July 2012 and August 2020. Patients with bAVMs were clinically diagnosed by neuroradiological data, and unruptured bAVMs were defined based on the combination of medical history and radiological findings as reported previously (13). All recruited patients had no clinical history of surgical, endovascular, or stereotactic radiosurgical treatment for bAVMs. Meanwhile, detailed clinical records were available for the evaluation of epilepsy. This study was approved by the ethics committee of Beijing Tiantan Hospital, Capital Medical University, and written informed consent was obtained from all patients. The flow chart of the study is illustrated in Figure 1.

### Evaluation of bAVM-Related Epilepsy

The diagnosis of epilepsy was clarified according to the classification and terminology of the International League Against Epilepsy based on the patient's observed behavior (14). Moreover, a patient was determined to have suffered from bAVM-related epilepsy when a history of at least one seizure with the presence of an enduring alteration (i.e., bAVM) in the brain was reported (15).

### Image Acquisition and Lesion Segmentation

The MR images were obtained using a 3.0-T MR scanner (SIEMENS Trio). Axial TOF-MRA images were obtained for all the patients. The sagittal T1 anatomical image acquired was a gradient-echo sequence: TR, 2,300 msec; TE, 2.98 msec; slice thickness, 1 mm; slices, 176; FOV, 256 mm; flip angle, 9°; matrix, 64 × 64; voxel size, 1 × 1 × 1 mm^3^; and bandwidth, 240. Axial TOF-MRA was performed using a three-dimensional (3D) TOF gradient-echo acquisition sequence: TR, 22 msec; TE, 3.86 msec; slice thickness, 1 mm; slices, 36 × 4; FOV, 220 × 220 mm^2^; flip angle, 120°; and matrix, 512 × 512 (16).

Masks of lesions were drawn on each patient's TOF-MRA image using 3D Slicer software (www.slicer.org) by two neurosurgeons (Y.M.J. and H.L.) who were blinded to the patients' clinical information. We delineated the lesion area according to the texture of light and dark reflected by vessels and tissues. The lesion masks were combined when there was less than a 5% discrepancy between the individual masks identified by the two neurosurgeons, and the masks we used were determined by a senior neurosurgeon (Y.C.) when the individual masks from the two neurosurgeons were inconsistent (>5%).

### Extraction of Quantitative Radiomic Features

Based on PyRadiomics, a total of 858 radiomic features were extracted, including 7 location features, 18 first-order statistics (FOS) features, 14 shape- and size-based features, 75 texture features, and 744 wavelet features. The location features were extracted based on the method applied in the research of Liu et al. (10). In brief, a coordinate system with the anterior commissure (AC) as the origin point was developed in the Montreal Neurological Institute (MNI) space and the lesion segmentation of each patient was registered to the MNI template. Based on the coordinate system, the polar coordinates (γ, θ, and Φ) of the centroid of the bAVM and the distance (Cityblock, Chebyshev, Mahalanobis, and Cosine distance) from the AC to the centroid of the bAVM were calculated. The FOS features reflected the distribution of voxel values within the 3D matrix of the region of interest and the overall information of the bAVMs. The shape- and size-based features reflected the volume, surface area, and shape of the lesions. The texture features describing the internal and surface textures of the bAVMs included 24 gray-level co-occurrence matrix (GLCM) features, 14 gray-level dependence matrix (GLDM) features, 16 gray-level run-length matrix (GLRLM) features, 16 gray-level size zone matrix (GLSZM) features, and 5 neighborhood gray-tone difference matrix (NGTDM) features. The wavelet features were calculated by FOS, GLCM, GLDM, GLRLM, GLSZM, and NGTDM features through Coiflet 1 3D wavelet transform.

### Feature Screening and Model Building

Epilepsy-related radiomic features were screened by the least absolute shrinkage and selection operator (LASSO) method, which is suitable for the regression of high-dimensional data. Meanwhile, multivariate logistic regression analysis was used to select independent predictors for epilepsy among clinical features. Three logistic regression models were constructed based on the screened features. The selected radiomics features were applied to build a radiomics model, and a radiomics score was calculated for each patient through a linear combination of the selected features weighted by their respective coefficients. A clinical model was developed based on the screened clinical features. Finally, a combined model was constructed by incorporating the significant variables of the clinical factors and the radiomics score.

### Assessment of the Performance of Different Models

Receiver operating characteristic (ROC) curve analysis based on 5-fold cross-validation was used to assess the model performance, and the area under the ROC curve (AUC) was calculated for quantification. Accuracy, sensitivity, specificity, positive predictive value, and negative predictive value were also used to assess the predictive performance. Additionally, calibration curves were plotted to assess the calibration of the combined model, accompanied by the Hosmer-Lemeshow test. Finally, to assess the clinical usefulness of the models, decision curve analysis (DCA) was performed by calculating the net benefits for a range of threshold probabilities in the whole cohort.

### Statistical Analysis

Statistical analyses of patients' baseline characteristics were performed with the SPSS statistical package (version 22.0.0). Continuous variables are summarized as the means ± SD, and categorical variables are summarized as frequency counts and percentages. Variables were compared between patients with or without epilepsy. Wilcoxon rank-sum tests, *t*-tests, and chi-squared tests were used as appropriate. Statistical tests were considered significant at *P* < 0.05, and variables with *P* < 0.05 in the univariate analysis were then used in the multivariate analysis. LASSO logistic analysis and figures with ROC curves were generated by Python (version 3.7). Nomogram construction, calibration plot construction, the Hosmer-Lemeshow test, and DCA were performed using R (version 4.0.2).

## Results

### Clinical Characteristics

Between July 2012 and August 2020, a total of 176 patients with unruptured bAVMs were included in this study. The baseline characteristics of all patients are summarized in Table 1. Of 176 patients, 100 (56.8%) suffered from epilepsy and 76 (43.2%) did not. The ages of patients with or without epilepsy were 26.6 ± 11.6 years and 30.7 ± 11.9 years (*P* = 0.023), respectively. The lesion size of patients with epilepsy was 46.5 ± 12.6 mm, and that of patients without epilepsy was 40.8 ± 12.1 mm (*P* = 0.003). The number of niduses involving the frontal lobe was 55 for patients with epilepsy and 20 for patients without epilepsy (*P* < 0.001). There were no significant differences in the other characteristics mentioned in Table 1 between patients with epilepsy and those without epilepsy.

**Table 1:** Demographic and clinical characteristics of patients.

**Characteristics**	**Epilepsy (*n* = 100)**	**No epilepsy (*n* = 76)**	***P*-value**
Age, (mean ± SD, years)	26.6 ± 11.6	30.7 ± 11.9	0.023^†^^¶^
Male, no. (%)	72 (72.0)	45 (59.2)	0.075^‡^
Size (mean ± SD, mm)	46.6 ± 12.6	40.8 ± 12.1	0.003^†^^¶^
Deep venous drainage, no. (%)	13 (13.0)	12 (15.8)	0.600^‡^
Left side, no. (%)	52 (52.0)	45 (59.2)	0.341^‡^
Lesion location, no. (%)^*^			
Frontal	55 (55.0)	20 (26.3)	<0.001^‡^^¶^
Temporal	31 (31.0)	20 (26.3)	0.497^‡^
Parietal	26 (26.0)	27 (35.5)	0.172^‡^
Occipital	14 (14.0)	18 (23.7)	0.099^‡^
Insula	5 (5.0)	4 (5.3)	1.000^‡^
**S-M Grading, no. (%)**			0.072^§^
I	10 (10.0)	12 (15.8)	
II	33 (33.0)	31 (40.8)	
III	44 (44.0)	26 (34.2)	
IV	11 (11.0)	5 (6.6)	
V	2 (2.0)	2 (2.6)	

*S-M Grading, Spetzler-Martin Grading*.

* *BAVMs involving more than one brain lobe were counted repeatedly*.

† *t-test*.

‡ *Chi-square test*.

§ *Wilcoxon rank-sum tests*.

¶ *P-value <0.05*.

### Feature Extraction and Model Construction

We extracted 12 clinical features and 858 quantitative radiomics features from a single patient. For clinical features, multivariable logistic regression analyses showed that patient age (*P* = 0.048), lesion size (*P* = 0.022), and frontal lobe involvement (*P* = 0.001) were independent risk factors for epilepsy (Supplementary Table 1). A clinical model was constructed based on these 3 clinical risk factors. Meanwhile, the radiomic model was constructed based on the 15 radiomics features screened by LASSO logistic analysis, including polar coordinates γ, polar coordinates Φ, W_LLH_.firstorder.Mean, W_LLH_.glcm.ClusterShade, W_LLH_.glcm.Correlation, W_LHL_.firstorder.Mean, W_LHL_.ngtdm.Strength, W_LHH_.firstorder.Mean, W_HLL_.gldm.DependenceVariance, W_HLH_.glszm.SmallAreaLowGrayLevelEmphasis, W_HHL_.glcm.Imc2, W_HHL_.glszm.SizeZoneNonUniformity Normalized, W_HHH_.firstorder.Skewness, W_HHH_.gldm.DependenceVariance, and W_HHH_.glszm.SmallAreaLowGrayLevelEmphasis. Finally, the combined model was constructed incorporating the clinical risk factors and the radiomics score calculated by the radiomic model.

### Comparison of Different Models

**Figure 2:**
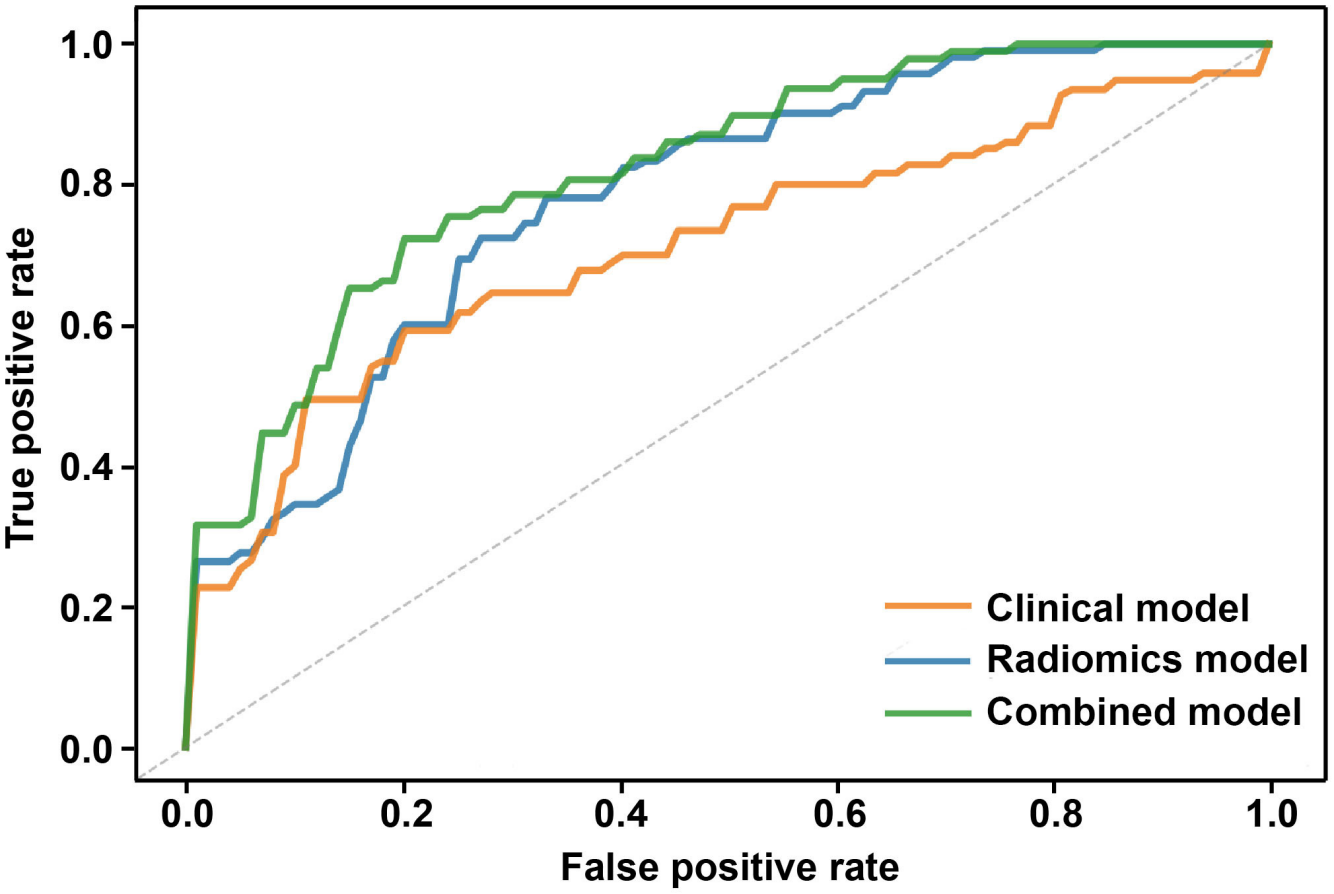
Comparison of ROC curves based on 5-fold cross-validation among the three models. The clinical model is marked with an orange curve, and the AUC is 0.71 (95% CI 0.62–0.80). The radiomics model is marked with a blue curve, and the AUC is 0.78 (95% CI 0.71–0.85). The combined model is marked with a green curve, and the AUC is 0.82 (95% CI 0.74–0.90).

ROC analysis was used to assess the ability of the 3 models to predict epilepsy. The ROC curves showed that the model built by the combination of clinical features and the radiomic score had favorable performance. The AUC for the combined model was 0.82 (95% CI 0.74–0.90), which was higher than that of the clinical model [AUC 0.71 (95% CI 0.62–0.80)] and radiomics model [AUC 0.78 (95% CI 0.71–0.85)] (Figure 2) Detailed results regarding the predictive performance of the 3 models are provided in Table 2.

**Table 2:** Diagnostic accuracy of prediction models.

**Models**	**AUC**	**SE**	**SP**	**PPV**	**NPV**	**ACC**
Clinical model	0.71	0.64	0.75	0.79	0.63	0.72
Radiomics model	0.78	0.72	0.74	0.78	0.69	0.73
Combined model	0.82	0.77	0.82	0.84	0.73	0.78

*ACC, accuracy; NPV, negative predictive value; PPV, positive predictive value; SE, sensibility; SP, specificity*.

### Clinical Use

**Figure 3:**
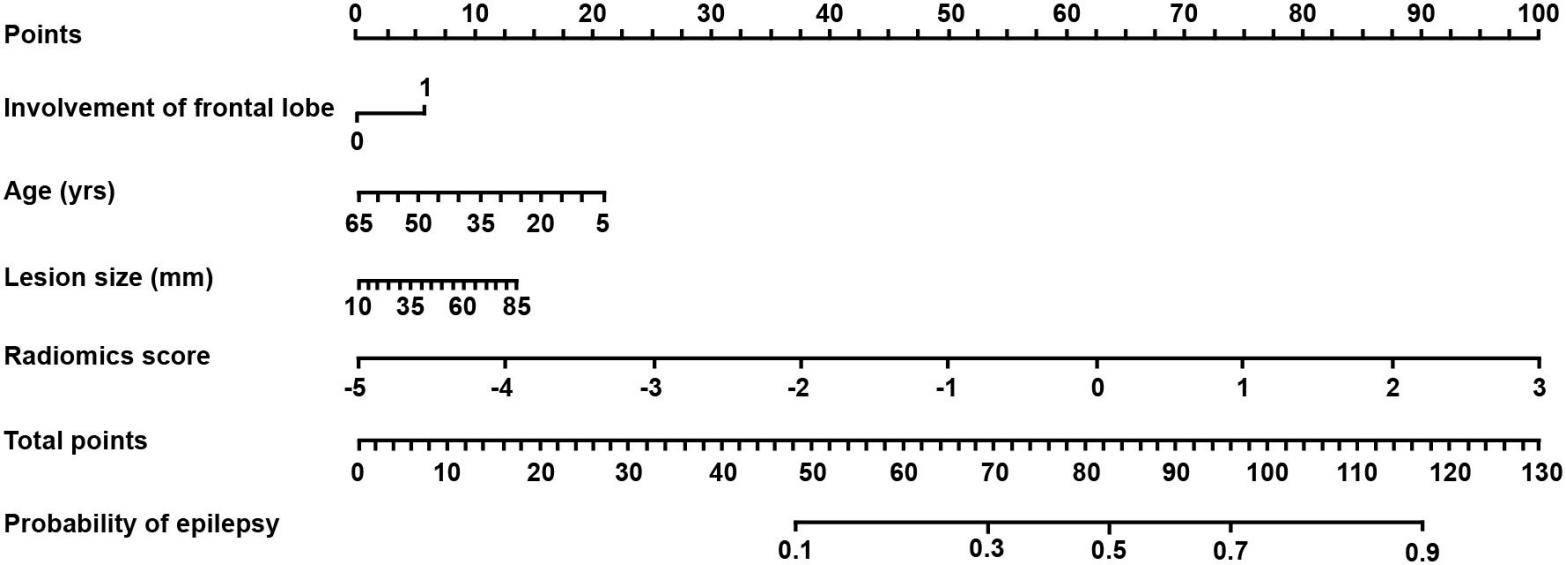
The radiomics-clinical nomogram derived from the combined model. The nomogram was built based on patient age, lesion size, involvement of the frontal lobe, and radiomics score. With the nomogram, the probability of epilepsy for each patient could be calculated on the basis of a logistic regression formula using the total points.

**Figure 4:**
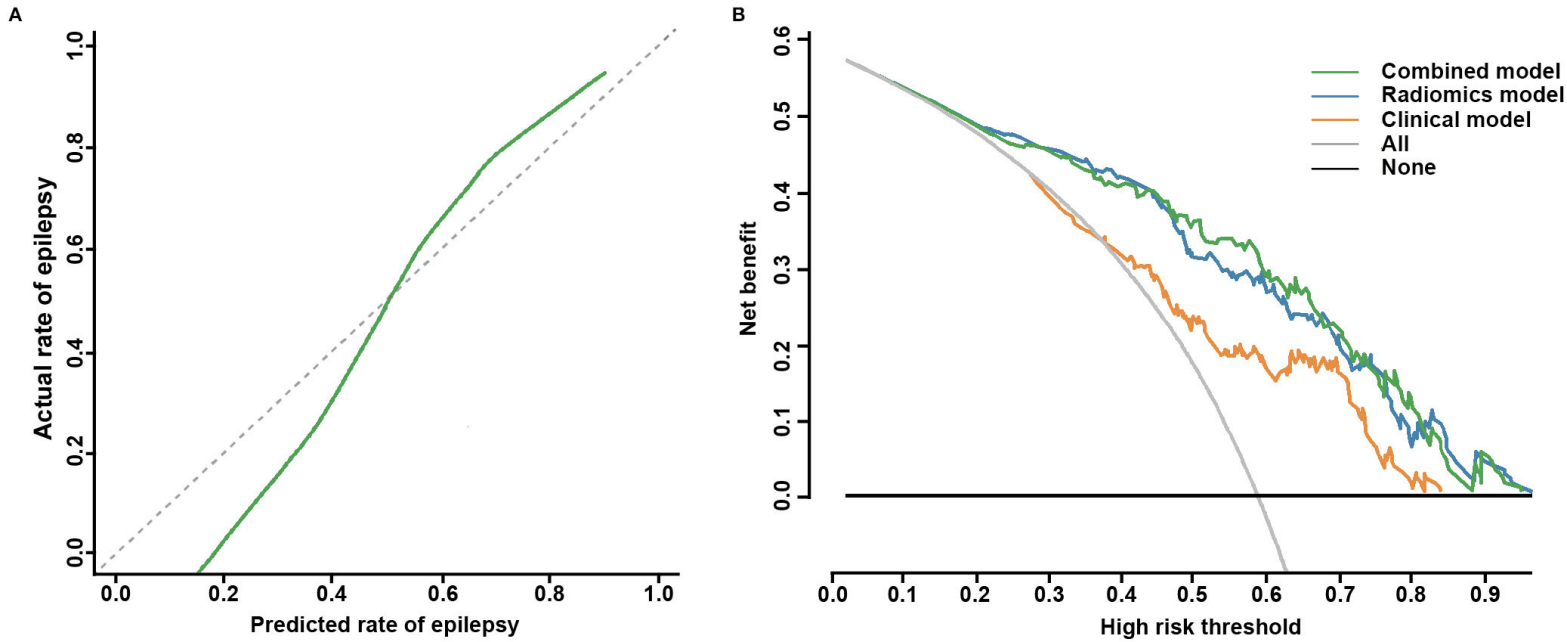
Calibration curve for the nomogram and decision curve analysis for the three models. **(A)** The calibration curve indicates the goodness-of-fit of the nomogram. The 45° gray dotted line represents the ideal prediction, and the green curve represents the predictive performance of the nomogram. The closer the green curve is to the ideal prediction line, the better the predictive efficacy of the nomogram. **(B)** Decision curve analysis of the three models in the whole cohort. The green curve, the blue curve, and the orange curve represent the net benefits of the combined model, the radiomics model, and the clinical model, respectively. The combined model had a higher net benefit than the other two models and simple diagnoses, such as all epilepsy patients (gray curve) or no epilepsy patients (black curve), across a large range of threshold probabilities at which a patient would be diagnosed with epilepsy.

Based on the combined model, a radiomics-clinical nomogram was developed for visualization (Figure 3). The calibration curve of the nomogram demonstrated favorable agreement between the ground truth and the predicted probabilities of epilepsy (Figure 4A). The Hosmer-Lemeshow test yielded non-significant statistics (*P* = 0.243), which suggested that there was no departure from the perfect fit.

The clinical utility of the 3 models was all evaluated, DCA was performed by quantifying the net benefits under different threshold probabilities. The DCA showed that the combined model had a higher overall net benefit in predicting epilepsy than the clinical model and the radiomics model across a large range of reasonable threshold probabilities, which demonstrated that the combined model had a relatively good performance in terms of clinical application (Figure 4B).

## Discussion

The accurate prediction of epilepsy could allow the customization of treatment options for patients with unruptured bAVMs. In this study, we developed and presented a quantitative and individualized epilepsy prediction model incorporating a series of clinical and TOF-MRA-based radiomics features associated with bAVM-related epilepsy. The results showed that the radiomics-clinical nomogram could successfully stratify patients according to their epilepsy risk. This easy-to-use nomogram may be a powerful clinical tool for assisting clinicians with personalized therapeutic decisions.

Recent developments in the field of radiomics have allowed the extraction of high-throughput imaging features, followed by automated analysis to assist clinical decision-making. Several studies have developed radiomics-based predictive models for various clinical characteristics, including survival outcomes (17), lymph node metastasis (18), and treatment responses (19). As epilepsy was among the important clinical characteristics associated with unruptured bAVMs, we applied the radiomics approach for the personalized prediction of epilepsy risk. Our results indicated that by incorporating the radiomics score and clinical characteristics, the risk of bAVM-related epilepsy could be evaluated with favorable accuracy.

Lesion location is an influential factor associated with bAVM-related epilepsy. Previous studies have shown that the frontal and temporal lobes are related to epilepsy in patients with bAVMs (4, 6). Moreover, at the brain subregion level, Zhang et al. found that the right precentral gyrus and the right superior longitudinal fasciculus tended to be epileptogenic, and the damage percentages to these regions of the lesions were considered location features and added into radiomic analyses for the prediction of bAVM-related epilepsy (9). Recently, a coordinate system was developed to quantitatively describe the location of brain lesions and was successfully applied to the prediction of tumor-related epilepsy by radiomics analysis (10, 20). In our study, we utilized this approach to obtain the location features of bAVMs. The polar coordinates based on the centroid of the bAVM and the distances from the AC to the centroid accurately described the lesion location, which provided more detailed information for the radiomic prediction models in the current study.

Studies of general epilepsy populations show that in 49–91% of patients with epilepsy, at least one seizure precipitant factor, including stress, lack of sleep, fatigue, emotions, and flickering lights, could be identified (21–24). It has been claimed that the prevention of seizure precipitants could improve the management of epilepsy (25–27). Therefore, patients with a high risk of epilepsy can benefit from behavior and lifestyle education if they could be screened out correctly with an imaging biomarker. This work is of great clinical significance considering that the radiomics-clinical nomogram showed favorable predictive efficacy in bAVM-related epilepsy, which could assist clinical treatment decision-making and achieve precision treatment.

The present study also has some limitations. First, given the stereotactic electroencephalographic data were not available, the diagnosis of the bAVM-related seizures was based on the clinical presentation. Second, the interpretability of radiomic features has remained an intractable task in the study of radiomics. Finally, although to our best knowledge, this study is the largest study termed patients with unruptured bAVMs, and our internal validation with a cross validation method showed favorable diagnostic performance, further external validation studies are needed to confirm our findings.

## Conclusion

The radiomics features extracted from TOF-MRA were associated with bAVM-related epilepsy. Combining the radiomics and clinical features, the radiomics-clinical nomogram could be a reliable tool for personalized treatment in patients with unruptured bAVMs, assisting in clinical treatment decision-making and achieving precision treatment.

## Data Availability Statement

The raw data supporting the conclusions of this article will be made available by the authors, without undue reservation.

## Ethics Statement

The studies involving human participants were reviewed and approved by the Ethics Committee of Beijing Tiantan Hospital, Capital Medical University. Written informed consent to participate in this study was provided by the patients/participants.

## Author Contributions

SZ designed the study, acquired the data, performed statistical analysis, and drafted the manuscript for intellectual content. QZ analyzed the data and revised the manuscript for intellectual content. YJ and HL masked the bAVMs on patients' TOF-MRA image and revised the manuscript for intellectual content. JWeng, RH, and JWang analyzed, interpreted the data, and revised the manuscript for intellectual content. HX and JZhang collected the data. YL, ZW, SW, and JZhao designed the study and revised the manuscript for intellectual. YC provided overall oversight of the research. All authors contributed to the article and approved the submitted version.

## Funding

This study was supported by the National Natural Science Foundation of China (81901175), the National Key Research and Development Program of China during the 13th Five-Year Plan Period (2016YFC1301803).

## Conflict of Interest

The authors declare that the research was conducted in the absence of any commercial or financial relationships that could be construed as a potential conflict of interest.

## Publisher's Note

All claims expressed in this article are solely those of the authors and do not necessarily represent those of their affiliated organizations, or those of the publisher, the editors and the reviewers. Any product that may be evaluated in this article, or claim that may be made by its manufacturer, is not guaranteed or endorsed by the publisher.

## Abbreviations

AC: Anterior commissure
AUC: Area under the receiver operating curves
bAVM: Brain arteriovenous malformation
DCA: Decision curve analysis
FOS: First-order statistics
GLCM: Gray-level co-occurrence matrix
GLDM: Gray-level dependence matrix
GLRLM: Gray-level run-length matrix
GLSZM: Gray-level size zone matrix
LASSO: Least absolute shrinkage and selection operator
MNI: Montreal Neurological Institute
NGTDM: Neighborhood gray-tone difference matrix
ROC: Receiver operating characteristic
TOF-MRA: Time-of-flight Magnetic Resonance Angiography.
