# Supplementary material for: Radiomics Analysis for Predicting Epilepsy in Patients With Unruptured Brain Arteriovenous Malformations
Source: Front Neurol. 2021 Dec 15;12:767165. doi: 10.3389/fneur.2021.767165 (PMC8714660; doi:10.3389/fneur.2021.767165)
Supplement: Supplementary file 1 [file Table_1.DOCX]

Supplementary Material

# Supplementary Table 1

| **Supplemental table 1. Univariable and multivariable analysis of risk factors associated with bAVM-related epilepsy**. | | | | | | | |
| --- | --- | --- | --- | --- | --- | --- | --- |
| Variables | Univariable | | |  | Multivariable | | |
|  | OR | 95% CI | P value |  | OR | 95% CI | P value |
| Age | 0.971 | 0.946-0.996 | 0.025^*^ |  | 0.972 | 0.946-1.000 | 0.048^*^ |
| Male | 1.771 | 0.941-3.334 | 0.076 |  |  |  |  |
| Size | 1.039 | 1.012-1.065 | 0.004^*^ |  | 1.031 | 1.004-1.059 | 0.022^*^ |
| Deep venous drainage | 0.797 | 0.341-1.862 | 0.600 |  |  |  |  |
| Left side | 0.746 | 0.408-1.363 | 0.341 |  |  |  |  |
| Frontal lobe involvement | 3.422 | 1.796-6.522 | <0.001^*^ |  | 3.175 | 1.631-6.179 | 0.001^*^ |
| Temporal lobe involvement | 1.258 | 0.648-2.443 | 0.498 |  |  |  |  |
| Parietal lobe involvement | 0.638 | 0.333-1.219 | 0.174 |  |  |  |  |
| Occipital lobe involvement | 0.525 | 0.242-1.137 | 0.102 |  |  |  |  |
| Insula lobe involvement | 0.947 | 0.246-3.654 | 0.937 |  |  |  |  |
| S-M grading |  |  |  |  |  |  |  |
| I |  |  | Reference |  |  |  |  |
| II | 1.277 | 0.483-3.376 | 0.621 |  |  |  |  |
| III | 2.031 | 0.770-5.353 | 0.152 |  |  |  |  |
| IV | 2.640 | 0.685-10.181 | 0.159 |  |  |  |  |
| V | 1.200 | 0.142-10.119 | 0.867 |  |  |  |  |
| Abbreviations: S-M Grading, Spetzler-Martin Grading; ^*^p<0.05. | | | | | | | |
